# Role of Probiotics in Stimulating the Immune System in Viral Respiratory Tract Infections: A Narrative Review

**DOI:** 10.3390/nu12103163

**Published:** 2020-10-16

**Authors:** Liisa Lehtoranta, Sinikka Latvala, Markus J. Lehtinen

**Affiliations:** Global Health & Nutrition Science, DuPont Nutrition & Biosciences, Sokeritehtaantie 20, FI-02460 Kantvik, Finland; sinikka.latvala@dupont.com (S.L.); markus.lehtinen@dupont.com (M.J.L.)

**Keywords:** probiotic, respiratory virus, respiratory tract infection, immune, infection, *Lactobacillus*, *Bifidobacterium*, microbiota

## Abstract

Viral respiratory tract infection (RTI) is the most frequent cause of infectious illnesses including the common cold. Pharmacological solutions for treating or preventing viral RTIs are so far limited and thus several self-care products are available in the market. Some dietary supplements such as probiotics have been shown to modulate immune system function and their role in reducing the risk and the course of RTIs has been investigated extensively within the past decade. However, the mechanism of action and the efficacy of probiotics against viral RTIs remains unclear. We searched PubMed, Google Scholar, and Web of Knowledge for pre-clinical and clinical studies investigating the effect of probiotics on respiratory virus infections, immune response, and the course of upper and lower respiratory tract illness. The literature summarized in this narrative review points out that specific probiotic strains seem effective in pre-clinical models, through stimulating the immune system and inhibiting viral replication. Clinical studies indicate variable efficacy on upper respiratory illnesses and lack proof of diagnosed viral infections. However, meta-analyses of clinical studies indicate that probiotics could be beneficial in upper respiratory illnesses without specific etiology. Further studies aiming at discovering the mechanisms of action of probiotics and clinical efficacy are warranted.

## 1. Introduction

Respiratory viruses cause the most common infectious illnesses in humans—acute RTIs that can be divided into upper RTIs (URTI), e.g., the common cold, and lower RTIs (LRTI), e.g., bronchitis and pneumonia. These illnesses affect all age groups annually and cause a high burden on health care systems and global economics due to absenteeism from daycares, school, and work. Over 200 virus types have been identified as causative agents for respiratory illnesses [1,2]. In most cases, especially illnesses of the upper respiratory tract are mild to moderate and often self-limiting. On the other hand, LRTIs leading to pneumonia can be especially fatal among children and the elderly, or immunocompromised subjects [2].

In the past decade, studies linking the microbiota and immune system function have laid the foundation for the opportunities in microbiota modulation and bacterial therapeutics in health and disease. Further, studies have associated the gut and airway microbiota with upper and lower respiratory tract health and immunity [3,4]. Thus, modulation of the gut microbiota and immunity by dietary supplements or pharmaceuticals is of increasing interest in finding novel solutions to manage RTIs. Among the promising candidates are probiotics that have been studied for immune function modulation and viral infections. Meta-analyses suggest that probiotics could be beneficial in the context of acute URTI typically caused by viruses [5,6].

We conducted a literature search with keywords “probiotics”, “*Lactobacillus*”, *Bifidobacterium*” “respiratory tract infections”, “viral respiratory tract infections”, and “respiratory virus” in common databases such as PubMed, Google Scholar, and Web of Knowledge up to March 2020. In this narrative review, we focus on evaluating the current knowledge in the scientific literature on the immunomodulatory effects of probiotics in the context of viral RTIs by reviewing key pre-clinical and clinical evidence.

## 2. Etiology and Epidemiology of Viral Respiratory Tract Infections

The most common viruses causing respiratory infections are picornaviruses (rhinoviruses and enteroviruses), coronaviruses, adenoviruses, respiratory syncytial virus (RSV), parainfluenza, and influenza viruses [1,7]. In recent decades, advancements in molecular diagnostics have also enabled the identification of several novel respiratory viruses, including human bocaviruses and metapneumovirus as well as highly pathogenic coronaviruses (severe acute respiratory syndrome: SARS-CoV, SARS CoV-2, and MERS-CoV) [8,9]. Estimations show that rhinoviruses that circulate in the community throughout the year cause approximately half of all the common cold cases, in contrast to seasonal viruses such as influenza and coronaviruses that cause approximately one-third of the common colds [1]. Individuals can also be infected simultaneously with multiple viruses.

Children tend to have more frequent infections than adults and the elderly due to their still maturing immune system [10]. The elderly, on the other hand, are more susceptible to severe complications due to aging-associated decline of the immune system function. The duration of the infection and presentation of symptoms, i.e., illness, varies between individuals [11]. Several respiratory viruses are detectable in asymptomatic subjects and, for instance, one-fifth of rhinovirus-infected individuals do not experience symptoms and do not feel ill despite carrying the rhinovirus [12]. On the other extreme, respiratory illnesses can last for weeks and be fatal, as in the case of emerging viruses, i.e., new influenza strains or coronaviruses capable of causing SARS. The underlying reason between these differences is due to genetic and epigenetic differences in immune responses and in the characteristics of the infecting virus, but potentially also the differences in the respiratory microbiota [13].

## 3. Immune Responses against Respiratory Viruses

Viruses that cause RTIs are found in various virus families, which differ in virulence and utilize variable strategies to infect the host cells and to evade the host immune system [14,15]. Respiratory viruses spread via nasal secretions that can be transmitted through the air or by hand-to hand and surface-to-hand contact [16]. Infection requires penetration of the virus through the host mucus layer, including the microbiota, and antiviral molecules in the mucus, such as antibodies and collectins. Once on the mucosal epithelial cells, respiratory viruses attach to specific receptors, such as Intercellular Adhesion Molecule (ICAM)-1 (rhinoviruses), peptidases (coronaviruses), or sialic acids (influenza viruses), that mediate the internalization of the virus by endocytosis [17,18]. The viral receptors are differentially expressed on host cells resulting in virus-specific host cell tropism that is one key factor in viral pathogenesis. For example, influenza viruses typically infect bronchial cells, whereas rhinoviruses infect the epithelial cells of the upper airways, resulting in differences in illness presentation.

Viral genomic structure can be, for example, positive- or negative-sense single-stranded (ss) or double-stranded (ds) RNA or DNA [19]. Most RTI causing viruses, picornaviruses, influenza viruses, and coronaviruses are ssRNA viruses, whereas adenoviruses are dsDNA viruses. The genomic structures are recognized by different receptors in the host and activate different types of immune responses. Some respiratory viruses, e.g., coronaviruses, are surrounded by a viral envelope which confers additional protection from the host immune system. Respiratory viruses have further developed molecules that help in evading the immune response, for example, by disrupting the interferon (IFN) response and hijacking the host’s cellular machinery for the production of virus copies [15].

Once the viruses have penetrated into the host cells, the epithelial and immune cells detect the viral structures by pattern recognition receptors (PRR) of which Toll-like receptors (TLRs) and retinoic acid-inducible gene I (RIG-I)-like receptors (RLRs) play a central role. TLR3, TLR7, TLR8, and TLR9 are located in the endosomes and can identify viral ss (TLR7 and 8) and ds (TLR3) RNA structures, and DNA (TLR9) [18]. The recognition by TLRs leads to activation of transcription factor nuclear factor kappa-light-chain-enhancer of activated B cells (NF-κB) and IFN regulatory factors (IRF) 3, 5, and 7 [12,20,21], resulting in expression of pro-inflammatory cytokines and type I IFNs, IFN-α and IFN-β. Type I IFNs are broadly secreted by cells, but epithelial cells further secrete type III lambda IFNs in response to viral infections. Cytoplasmic RNAs, on the other hand, are recognized by RLRs, of which RIG-I recognizes ssRNA and melanoma differentiation associated 5 (MDA-5) dsRNA. The activation of RLRs leads to type I (and type III in epithelial cells) IFN production via mitochondrial antiviral signaling protein (MAVS). Type I and III IFNs induce an antiviral state in the surrounding cells which is not, however, necessarily sufficient to resist the infection, but delays the spreading of the infection [12,22]. The activation of RLRs, and TLRs by viral infection and cellular stress, leads to formation of nucleotide-binding oligomerization domain (NOD)-, leucine-rich repeat (LRR)-, and pyrin domain-containing protein 3 (NLRP3) inflammasome [23]. Although the role of NLRP3 is still somewhat unclear in viral RTIs, it seems to play a role at least in rhinovirus, influenza, adenovirus, and RSV infections. NLRP3 inflammasome activation drives caspase 1-dependent IL-1β and IL-18 cytokine response and inflammatory programmed cell death (pyroptosis) [24].

Epithelium-derived pro-inflammatory cytokines tumor necrosis factor (TNF)-α, interleukin (IL)-1β, IL-6, chemokine (C-C motif) ligand (CCL) 2, CCL5, chemokine (C-X-C motif) ligand (CXCL)8, and CXCL10 induce innate cellular responses by attracting and activating Natural Killer (NK) cells, macrophages, and neutrophils that further amplify the innate cytokine and chemokine response [17]. The role of other innate cells like, for example, intraepithelial lymphocytes, γδT cells, mucosa associated invariant T cells (MAIT), and innate lymphoid cells (ILC) is less well described in viral infections, however, they are likely to contribute to innate and adaptive responses against viral infections, as exemplified by the NK cells [25,26] and by the role of ILC2 cells in overcoming an influenza infection [27]. If the innate immune or memory responses cannot clear the pathogen effectively and the adaptive immune system is unexperienced with the virus, an adaptive immune response is initiated and required. Key are dendritic cells (DCs) that present the viral antigens and induce B and T cell responses against the pathogen in the secondary lymph nodes. B and T cell responses initiate within four–six days post-infection and peak later at days 7–14 depending on the respiratory virus [28,29,30]. Typically, common respiratory viruses, such as rhinovirus and influenza virus, are cleared before adaptive immune responses are activated [22,30] indicating that memory responses and innate immunity are essential in viral eradication. However, the induction of cytotoxic CD8 T cells, CD4 T cells, and antibody responses is key for virus eradication by adaptive immunity and for establishing protective immunity for secondary infections.

The activation of the epithelium, innate immune cells, and adaptive responses is important for defense against respiratory viruses, but on the other hand, the host inflammatory response is the major cause of symptoms and more severe pathologies [12,18,31]. Chronic activation of CD8 T cell responses and adaptive immunity may lead to pulmonary damage and acute respiratory distress syndrome, like in severe cases of coronavirus infections (e.g., SARS-CoV or SARS CoV-2) and pandemic influenza virus infections [18,29,32]. In milder colds, rhinoviruses are not cytolytic and do not actually cause considerable damage to host cells and may pass asymptomatically. Presentation of cold symptom severity seems to correlate with host inflammatory response. Specifically, the early expression of pro-inflammatory IL-8 [33] and high levels of neutrophils in nasal aspirates [34] have been shown to correlate with symptom severity of rhinovirus and influenza infection [35,36]. Production of anti-inflammatory IL-10, resolvins, and regulatory T cell responses acts as a natural mechanism to control lung inflammation during acute influenza virus (and others) infection [37,38,39]. Virus–host immune interactions are key to viral pathogenesis and to ultimately determine the outcome of the infection.

## 4. Probiotics and Immune Modulation in Viral Respiratory Infections

### 4.1. Overview of Probiotics and Immunomodulatory Mechanisms

Probiotics, by definition, are live microorganisms that, when administered in adequate amounts, confer a health benefit on the host [40]. Most probiotics are lactic acid bacteria, belonging to *Lactobacillus* spp., (now with new taxonomy including *Lacticaseibacillus* spp., *Lactiplantibacillus* spp., *Levilactobacillus* spp., *Ligilactobacillus* spp., *Limosilactibacillus* spp. [41]) or *Bifidobacterium* spp. Furthermore, some strains of other microbial genera, such as *Propionibacterium* spp., and *Bacillus* spp., have been reported to have probiotic properties. Traditional lactic acid bacteria have long been considered safe and suitable for human consumption as very few instances of infection have been associated with these bacteria, and several published studies have specifically addressed their safety (reviewed, e.g., by [42,43,44]). Regarding probiotics safety in RTIs, meta-analyses conclude that the reported side effects related to the consumption of probiotics were minor [5,6,45].

Probiotics are mostly consumed orally in the form of dietary supplements and food (e.g., yoghurt). Therefore, their primary site of action is in the gastrointestinal (GI) tract [46]. However, probiotics have been detected with PCR-based methods from nasopharyngeal mucosa, adenoids, and tonsils after oral consumption [13,47,48], but it is unclear what the contribution to upper respiratory tract immune stimulation against viral RTI by probiotics is, as oral ingestion results in the stimulation of the intestinal immune system as well. The small intestine, that is naturally exposed to microbes and nutrients due to the thin mucosal layer, seems to play an important role in immune stimulation by probiotics [49,50]. However, dissecting the relative contributions of the small and large intestine and upper GI tract on immune stimulation against RTI is challenging with available research data. Independent of the actual mucosal inductor site of the probiotic, it has been shown that lymphocytes circulate between mucosal tissues [51]. Thus, local mucosal stimulatory effects may influence immune responses at other mucosal tissues and contribute to antiviral immunity.

Even very closely related bacteria have differences in their antigenic structures and thus influence the immune system uniquely. Probiotics are thought to influence immune function primarily in a strain-specific manner [40]. Although these effects in general are strain-specific, probiotics also share common mechanisms of immune stimulation, such as the secretion of metabolites. For instance, short chain fatty acids are known to have immunomodulatory effects [52]. Direct effects of the probiotics on immune function are driven by interactions of bacterial structures or metabolites with receptors, like TLRs, on the host epithelial and immune cells. On the other hand, probiotics may influence immune function indirectly by changing the composition and/or activity of the host microbiota [53]. For example, in the small intestine where the number of endogenous bacteria is lower than in the large intestine, ingestion of probiotics temporally changes the microbiota composition and influences the host immune response [49,50]. In RTIs, orally consumed probiotics may elicit systemic effects from the GI tract via the “gut–lung axis” by modulating mucosal immune function [54,55]. Probiotics are taken up by M cells or by CX3C chemokine receptor 1 (CX3CR1)+ macrophages located in the gut epithelium and then transferred to DCs in the subepithelial tissue. Probiotics are able to modulate DC polarization and function [56] that influence the subsequent T and B cell responses [57] in the inductive sites (Peyer’s patch, and mesenteric lymph nodes). T and B cells can also enter the circulation and migrate to extraintestinal sites, such as the respiratory tract [51,58]. However, the exact mechanisms of probiotics (and their metabolites) in respiratory infections has not been clearly established and may be influenced by the investigated probiotic strain, microbiota composition, and immunological status of an individual. In the following chapters, we review the current pre-clinical evidence of immunomodulatory and antiviral mechanisms of probiotics against respiratory virus infections with a focus on the direct stimulatory effects of probiotics on virus-infected immune cells and animal models.

### 4.2. Probiotic Immunostimulation and Inhibition of Viral Replication In Vitro

Probiotic bacteria can engage and activate TLRs leading to the activation of NF-κB and IRFs in immune cells that are essential in antiviral defense. For example, it has been demonstrated in murine bone marrow-derived DCs that *Lactobacillus acidophilus* NCFM and *L. acidophilus* X37 induce the upregulation of TLR3, IL-12, and IFN-β in a TLR2-dependent manner [59]. In macrophage-derived RAW264.1 cells, *Lactobacillus gasseri* SBT2055 upregulated IFN-β and myxovirus resistance (Mx)1 mRNA expression [60] and in human monocyte-derived macrophages, two *Lacticaseibacillus rhamnosus* strains, GG and LC705, induced type I IFN-dependent gene activation [61]. Pro-inflammatory and IFN-regulated genes including IL-6, IL-12, IL-1β, IL-8, CCL20, CXCL10, Mx1, and Mx2 were induced by both *Lacticaseibacillus* strains. In addition, the gene expression of TLR3 and TLR7, receptors recognizing viral dsRNA and ssRNA, respectively, was upregulated by these strains. TLR3 gene expression was upregulated only by *L. rhamnosus* LC705, the strain with higher antiviral potential, while TLR7 was moderately upregulated by both strains.

In vitro studies with immune cells have also shown the ability of probiotics and their components to restrict viral replication. In human monocyte-derived macrophages, *Lacticaseibacillus* strains showed the ability to prevent influenza A virus replication which correlated with the ability to activate type I IFN-dependent antiviral genes [61]. Similarly, mouse adapted influenza A virus (PR8) titer was reduced in RAW264.1 cells by *L. gasseri* SBT2055 [60]. In mouse bone marrow DCs, the inhibition of viral replication by *L. acidophilus* ATCC4356 S-layer protein was demonstrated [62,63]. Priming of cells with S-layer protein prior to H9N2 avian influenza virus infection inhibited the invasion and replication of the virus, stimulated the type I IFN signaling pathway, increased IL-10 mRNA, and decreased TNF-α mRNA expression [62].

Polyinosinic/polycytidylic acid (Poly I/C), a synthetic mimic of dsRNA, is widely used in in vitro studies to stimulate TLR3. It induces characteristic inflammatory responses associated with virus infections such as increased production of inflammatory cytokines. Stimulation of airway epithelial cells with Poly I/C has been found to closely mimic inflammatory responses associated with respiratory virus infections [64]. Poly I/C challenge in peripheral blood mononuclear cells (PBMCs) induced changes in the gene expression of TLR3, IFN, and NF-kB-dependent pathways similar to acute viral infections [63]. Moreover, Poly I/C was able to induce a pulmonary dysfunction similar to RSV in a mouse model [65].

Probiotic bacteria have shown the ability to modulate Poly I/C-induced responses. Studies with heat-killed *Lacticaseibacillus casei* CRL431 showed that the strain reduced TNF-α, IFN-γ, and IL-17 levels when it was introduced before or simultaneously with Poly I/C challenge in PBMCs alone and in a co-culture system with alveolar epithelial cell line A549 [66]. IL-10 and IL-29 (also known as IFN-λ1) were induced in response to Poly I/C together with heat-killed *L. casei* CRL431, indicating a boost in pro-inflammatory responses and the activation of anti-inflammatory and antiviral mechanisms. In the intestinal human colon cell line (HCT116), regulation of Poly I/C response by *Lactiplantibacillus plantarum* subsp. *plantarum* DU1, *Latilactobacillus sakei* DU2, and *Weissella cibaria* DU1 was examined [67]. These strains modified Poly I/C-induced expression of cytokines and antiviral genes by upregulating IFN-β, TLR3, and RIG-I while dampening the inflammatory response. Moreover, the probiotic strains induced IFN-α, IFN-β, and IL-10 and reduced the expression of inflammatory cytokines IL-1β and TNF-α in human monocytic THP-1 macrophages [67]. In addition to the pro-inflammatory and antiviral gene activation described above, also direct interactions of viruses and probiotic bacteria have been demonstrated between porcine influenza A virus and *Enterococcus faecium* in vitro [68]. Similar upregulation of IFN response by probiotics has been shown in several studies in intestinal epithelial cells [69,70,71] and macrophages [68,72]. Overall, the in vitro studies indicate that probiotics may stimulate similar innate immune pathways to respiratory viruses and potentially modulate virus-induced immune responses.

### 4.3. Antiviral Effects of Probiotics in Animal Studies

Several mouse studies have shown that the administration of probiotics can help to fight against viral RTIs. The beneficial effects of oral probiotic supplementation on mouse survival and health status is well demonstrated [60,73,74,75,76,77]. For example, oral administration of *Lacticaseibacillus paracasei* subsp. *paracasei* CNCM-I-1518 [74], *L. gasseri* LG2055 [60], and *Bifidobacterium longum* MM-2 [75] reduced mortality and improved immune control in influenza-infected mice. Probiotic administration resulted in better health status of the mice and lower virus loads in the lungs after influenza infection. In addition, the immune response to viruses was modulated by probiotic administration. For instance, *L. paracasei* CNCM-I-1518 modified the pro- and anti-inflammatory cytokine release in the lungs before and after influenza infection and affected total cell counts in the lungs after probiotic treatment [74]. Similarly, *B. longum* MM-2 suppressed inflammation in the lower respiratory tract through the decrease in influenza virus proliferation and pulmonary IL-6 and TNF-α cytokine production [75]. Activation of host defense systems by increased IFN-γ, IL-2, IL-12, and IL-18 gene expression and NK cell activation in lungs was also demonstrated. In non-infected mice, *B. longum* MM-2 significantly enhanced IFN-γ production by Peyer’s patch cells and splenic NK cell activity. In the infected mice, NK cell activity was significantly enhanced both in the spleen and lungs by the probiotic strain. Another *Bifidobacterium* (*B. bifidum*) improved anti-influenza immune responses by inducing both humoral and cellular immunities [76]. Decreased IL-6 levels were detected in the lung and higher IgG1 and IgG2 levels in the sera of probiotic-treated mice compared with control mice. Furthermore, *L. gasseri* SBT2055 has been found effective in preventing both influenza A virus [60] and RSV [78] infections in mice. Pre-treatment with *L. gasseri* SBT2055 induced the expression of antiviral genes Mx1 and 2′-5′ oligoadenylate synthase (OAS)1a in lung tissues before viral infection and reduced lung inflammatory responses after viral infection [60]. The same *L. gasseri* strain was found effective in preventing RSV infection in mice by reducing lung viral loads and pro-inflammatory cytokines and by stimulating IFNs and IFN responsive gene expression such as IFN-β1, IFN-γ, interferon-inducible transmembrane protein (IFITM)3, OAS1a, and interferon-stimulated gene (ISG)15 [78].

In addition to live probiotics, also orally administered heat-killed bacteria seem to confer protection against viral RTIs in mice [73]. Heat-killed *L. paracasei* MCC1849 reduced symptom scores and lung virus titers in influenza-infected mice and induced antigen-specific IgA production in the small intestine, serum, and lungs. The proportion of IgA+ B cells and follicular helper T cells (Tfh) in Peyer’s patches was increased as was the gene expression of IL-12p40, IL-10, IL-21, signal transducer and activator of transcription (STAT)4, and B cell lymphoma protein (Bcl)-6, which are associated with Tfh cell differentiation.

To conclude, different probiotic strains have been shown effective in inhibiting the replication of various respiratory viruses including influenza viruses and RSV in vitro. Similar effects have been demonstrated in several mouse studies with the ability to reduce virus titers in lung tissues and modulation of antiviral and pro-inflammatory gene expression before and after viral infection.

## 5. Clinical Evidence

Accumulating clinical evidence suggests that probiotics in general may have favorable effects against RTIs. For instance, several systematic reviews and/or meta-analyses have evaluated the effects of prophylactic ingestion of probiotics on the RTI-associated outcomes, e.g., either only in children [79,80], or both in children and adults [5,6,45] (Table 1). Of note, the majority of the outcomes in these analyses are related to URTI, and data on LRTI outcomes are either not available or are very limited. Therefore, in the below chapter, we primarily focus on clinical trials on probiotics’ effects on URTI symptoms/episodes/duration.

In children (below 18 years), the meta-analysis by Wang et al., 2016, reported that probiotic use compared with placebo significantly decreased the number of subjects having at least one RTI episode, had fewer numbers of days of RTIs per person, and had fewer numbers of days absent from daycare or school [80]. However, the meta-analysis did not find a statistically significant difference on the illness episode duration between the probiotic and the placebo. Laursen and Hojsak [79] limited the analysis to children up to 7 years old and reported that probiotic use was associated with reduced risk of at least one URTI and reduced the risk of antibiotic use, but the use was not associated with a reduction in RTI duration or missed days of daycare due to RTI [79]. This meta-analysis also discussed the effects of the individual probiotic strains on RTI outcomes. The results of the analysis showed that the most effective probiotic strains on RTI-related outcomes were *L. rhamnosus* GG (RTI duration) and *L. acidophilus* NCFM as a single supplement and in combination with *B. lactis* Bi-07 (RTI duration and antibiotic use). Interestingly, these strains have shown in vitro the ability to induce antiviral IFN signaling pathways (see Section 4.2) which may potentially explain their beneficial effects observed in RTIs. However, as multiple studies with probiotic strains other than *L. rhamnosus* GG are limited or lacking, comparison and interpretation of the strain specific results should be made carefully.

Meta-analyses that pool data from clinical trials conducted with children, adults, and the elderly show that probiotic use is more beneficial over placebo in reducing the number of participants experiencing episodes of acute URTI [5,6], reducing antibiotic prescription rates for acute URTIs [5,6], and reducing the mean duration of an episode of an acute URTI as well as cold-related school absences [5,45]. When the literature search was conducted, meta-analyses were not found in the databases searched on probiotic effects on respiratory infections restricted to the elderly population, potentially due to the fact that data are fairly limited regarding this age group.

While there is consensus that probiotics could have potential in reducing the risk for RTIs, it should be noted that clinical trials in the meta-analyses have been conducted in populations of different ages and genetic backgrounds, with various strains and/or their combinations, supplementation matrices, and doses. Moreover, the measured outcomes and data collection procedures between the trials (i.e., infection episode definition) are not harmonized and therefore may vary considerably. Consequently, pooling all the data creates a bias, as the probiotic effect is generally dependent on the dose, population, and strain. Moreover, as discussed above, the probiotics effects on the immune system are strain-specific which affects the interpretation of the results.

With regard to probiotics effects to specific respiratory viruses in clinical settings, several trials have characterized the respiratory infection etiology in infants [81], in children [82,83,84], in adults [85], and in the elderly [86]. In addition, two clinical trials have investigated the efficacy of probiotics in an experimental rhinovirus challenge model [87,88,89] (Table 2).

In the clinical trials conducted in free-living subjects in the community, no consistent data exist that show that specific probiotics would reduce the incidence of laboratory-confirmed respiratory virus infections as such. In preterm infants, the use of *L. rhamnosus* GG for 60 days was associated with lower incidence of rhinovirus-induced episodes (comprising 80% of all RTI episodes) compared with the placebo. However, *L. rhamnosus* GG had no effect on rhinovirus RNA load during infections, duration of rhinovirus RNA shedding, duration or severity of rhinovirus infection, or the occurrence of rhinovirus RNA in asymptomatic infants. In children attending daycare, *L. rhamnosus* GG [83] consumption for 28 weeks did not reduce the occurrence of any of the common respiratory viruses either. In otitis-prone children, supplementation of a combination of *L. rhamnosus* GG, *L. rhamnosus* Lc705, *B. breve* 99, and *Propionibacterium jensenii* JS, for six months, reduced the number of human bocavirus-positive nasopharyngeal samples when compared with placebo, but not the number of rhino/enterovirus-positive samples [84]. Furthermore, in schoolchildren, the consumption of *Levilactobacillus brevis* KB290 during influenza season was associated with lower incidence of physician-diagnosed influenza virus cases [82]. In adults attending military service, the use of a combination of *L. rhamnosus* GG and *B. lactis* BB-12 for either 90 or 150 days was not overall associated with lower occurrence of common respiratory viruses upon presentation of cold symptoms [85]. However, in a subgroup, there was a lower occurrence of rhino/enteroviruses after three months in the probiotic group when compared with the placebo. In nursing home residents, Wang et al., 2018, reported that the use of *L. rhamnosus* GG for six months was not associated with the reduction in occurrence of confirmed viral respiratory infections [86]. The differences between the findings in these trials may be explained by the fact that these studies were conducted in various age groups with different immune system statuses (infants vs. children vs. healthy adults vs. the elderly), different seasons, as well as variable probiotic strains, strain combinations, doses, and variable lengths of intervention. Furthermore, most of the studies were not designed for analyzing the viral infection etiology as the primary outcome and the diagnosis for the identification of the viral agent was not applied.

Since over 200 respiratory virus types can cause respiratory infections and, in many cases, the infections and symptoms overlap, or the etiology is undiagnosed, the potential antiviral effects of probiotics against specific viruses can be difficult to determine in clinical trials targeting free-living subjects within the community. To overcome this caveat, two probiotics have been investigated in an experimental rhinovirus challenge model that allows investigation of the effect of a probiotic strain to a specific viral pathogen. In a rhinovirus (type 39) challenge model, *B. lactis* Bl-04 was administered for 28 days prior and during five days of experimental rhinovirus infection to healthy volunteers [89]. *B. lactis* Bl-04 supplementation resulted in significantly lower rhinovirus titers in nasal washes during the infection as well as in a lower number of infected participants shedding the virus compared with the placebo. Moreover, *B. lactis* Bl-04 induced a significantly higher concentration of IL-8 in nasal washes after 28 days of supplementation and prior to infection. Given the reduced viral titer, an increase in IL-8 could indicate priming of the mucosal immune system prior to infection. This hypothesis is in line with a clinical study conducted in healthy active adults, where supplementation of *B. lactis* Bl-04 reduced the risk of URTI episodes compared with placebo [90]. In another similar experimental rhinovirus type 39 challenge pilot trial, no significant antiviral effect was seen with live or inactivated *L. rhamnosus* GG supplementation compared with placebo [87,88], suggesting potential strain-specific differences on the efficacy of probiotics in respiratory virus infections. Nevertheless, further adequately powered trials with harmonized study designs are necessary to draw conclusions on the efficacy of probiotics against specific respiratory viruses.

## 6. Discussion and Conclusions

**Figure 1:**
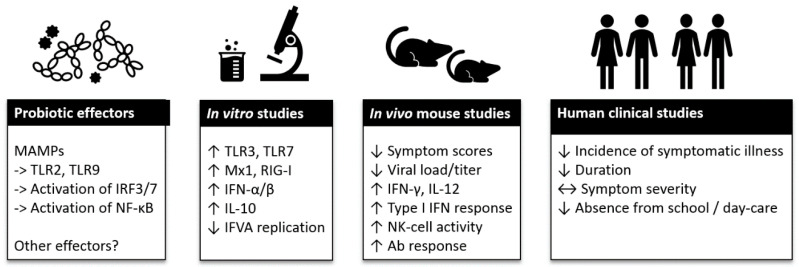
Summary of probiotic effector mechanisms and clinical evidence on viral respiratory tract infections. Abbreviations: Ab: antibiotic; IFN: interferon; IFVA: influenza A virus; IL: interleukin; IRF: interferon regulatory factor; NF-κB: nuclear factor kappa-light-chain-enhancer of activated B cells; MAMPs: microbe-associated molecular patterns; Mx: myxovirus-resistance protein; NK: Natural Killer; RIG: retinoic acid-inducible gene; TLR: Toll-like receptor. Symbols: ->: activation; ↑: increase; ↓: decrease; ↔: no effect.

Viral RTIs are the most common infections of mankind and the health and financial impact of seasonal epidemics and global pandemics on society is high. Due to the large number of various respiratory viruses, the development of efficient therapies, such as vaccines, is challenging. When preventative measures are scarce or lacking, the role of a well-functioning immune system becomes crucial for providing resistance to an infection. Within the past decade, research highlighting the importance of the microbiota on immune system function has raised interest in understanding the role of microbiota modulation and bacterial therapeutics by dietary and pharmaceutical solutions in health and disease. Of the available solutions, probiotic bacteria have been studied for immune function modulation in the context of respiratory viral infections. In this review, we have summarized the current evidence on the effects of probiotics on antiviral immune function in vitro and in vivo, and clinical evidence on the effect of probiotics on viral RTIs and on the course of RTI (Figure 1).

In vitro data indicate that probiotics have strain-specific immunomodulatory effects on the host and immune cells by engaging TLRs that stimulate IFN pathways. The upregulation of IFN response seems to prime cells for better resistance against virus infection as probiotics were shown effective in inhibiting the replication of various respiratory viruses, including influenza viruses and RSV. Similar effects have been demonstrated in mice with the ability of the probiotics to reduce virus titers in lung tissues and to modulate antiviral and pro-inflammatory gene expression before and after viral infection. Interestingly, some studies in mice show an increase in IL-10 response, suggesting control of the pro-inflammatory response that typically drives lung pathology in severe infections. Most likely probiotics’ effects in the gut are transferred into the respiratory tract via the gut–respiratory tract axis, however, this mechanism of action remains to be studied in more detail. The pre-clinical studies further show improvement in the symptom scores of mice, suggesting potential clinical benefits. Indeed, some evidence exists for specific probiotic strains, e.g., from the species of *L. rhamnosus*, *L. acidophilus,* and *B. lactis* for their ability to induce antiviral immune responses in pre-clinical models, which is in agreement with their effects observed in clinical trials in reducing the risk of RTI-associated outcomes. However, translation of probiotic effects from cell culture and animal studies to humans can be challenging and variable confounding factors, e.g., age, diet, microbiome, genetic and epigenetic immune status of an individual, study season, and variable viral epidemiology, all have an impact on the study outcome and are difficult to standardize. The clinical studies that have diagnosed and characterized viral etiology are limited, nevertheless, the meta-analyses investigating probiotic clinical interventions on RTIs show that probiotic use is associated with lower incidence and duration of mild RTIs, both in children and in adults. Further studies aiming at discovering the mechanism of action of probiotics and establishing the association of immune system function stimulation and clinical efficacy are warranted.

## Figures and Tables

**Table 1:** Systematic reviews and/or meta-analyses on the effects of probiotics on the respiratory tract infection-associated outcomes.

Study Type and Reference	No. of Included Studies for Meta-Analysis and Analysis Population	Probiotic Effect Compared with Control			
		RTI Incidence/Risk	RTI Duration	Absence from Daycare/School/Work	Antibiotic Use
Children					
Systematic review and meta-analysis	23 randomized, double-blinded, and placebo-controlled trials	Decreased number of subjects having at least 1 RTI episode (17 RCTs, 4513 children, relative risk 0.89, 95% CI 0.82–0.96, *p* = 0.004)	No significant difference of illness episode duration between study groups (9 RCTs, 2817 children, (MD 0.60, 95% CI 1.49–0.30, *p* = 0.19).	Fewer numbers of days absent from daycare/school (8 RCTs, 1499 children, MD 0.94, 95% CI 1.72–0.15, *p* = 0.02)	NA
[80]	6269 children (0–18 years)		Fewer numbers of days of RTIs per person (6 RCTs, 2067 children, MD 0.16, 95% CI 0.29–0.02, *p* = 0.03)		
Systematic review and meta-analysis	15 randomized placebo-controlled trials	Lower number of children with RTI, reduced RTI risk, 5 RCTs, *n* = 1841, RR 0.78, 95% CI 0.63–0.98, random effect model).	Probiotic consumption had no effect on the duration of RTIs (9 RCTs, *n* = 3529, MD -0.81 days, 95% CI −1.88–0.25, random effect model).	No effect on the days absent from daycare centers (9 RCTs, *n* = 3040, MD -0.25 days, 95% CI −0.75–0.24, random effect model).	Reduced the risk of antibiotic use (7 RCTs, *n* = 2858, RR 0.69, 95% CI 0.49–0.95, random effect model).
[79]	5121 children in daycare settings (3 months to 7 years)	No effect on the risk of at least one URTI (5 RCTs, *n* = 1711, RR 0.81, 95% CI 0.62–1.05, random effect model)			
All Age Groups					
Cochrane systematic review and meta-analysis	10 randomized, placebo-controlled trials	Lower number of participants experiencing episodes of acute URTI by 42–47%: (at least 1 episode: OR 0.58; 95% CI 0.36–0.92, *p* = 0.022; at least 3 episodes: OR 0.53; 95% CI 0.36–0.80, *p* = 0.002).	No efficacy when measuring the mean duration of acute URTI episode: MD −0.29; 95% CI −3.71–3.13 (*p* = 0.87)	NA	Reduced antibiotic prescription rates for acute URTIs: OR 0.67; 95% CI 0.45–0.98, *p* = 0.04
[6]	3451 participants (infants to elderly)	Reduction in episode rate ratio of acute URTIs (events per person/year) (rate ratio 0.88; 95% CI 081 to 0.96, *p* = 0.004)			
Cochrane systematic review and meta-analysis	12 randomized, placebo-controlled trials	Lower number of participants experiencing episodes of acute URTI by 47% (at least 1 episode: OR 0.53; 95% CI 0.37- 0.76, *p* value < 0.001; at least 3 episodes: OR 0.53; 95% CI 0.36–0.80, *p*= 0.002).	Reduced the mean duration of an acute URTI episode by 1.89 days (MD -1.89; 95% CI −2.03–1.75, *p* < 0.001)	Reduced cold-related school absence (OR 0.10; 95% CI 0.02–0.47 (only one trial)	Reduced antibiotic prescription rates for acute URTIs (OR 0.65; 95% CI 0.45–0.94, *p* = 0.024)
[5]	3720 participants (children to elderly)	No effect when measuring episode rate ratio (events per person/year) of acute URTI (rate ratio 0.83; 95% CI 0.66–1.05, *p* = 0.12)			
Systematic review and meta-analysis	20 randomized controlled trials	Reduced numbers of days of illness per person (standardized MD -0.31 (95% CI −0.41 to −0.22, *p* < 0.001)	Shortened illness episodes by almost a day (weighted MD -0.77 (95% CI −1.50 to −0.04), *p* = 0.04) (without an increase in the number of illness episodes)	Reduced numbers of days absent from daycare/school/work (standardized MD -0.17 (95% CI −0.31 to −0.03. *p* = 0.02),	NA
[45]	3-month-old children to elderly (participant numbers not specified)				

Abbreviations: CI; confidence interval; NA: not assessed/reported; MD: mean difference; OR: odds ratio; RCT: randomized controlled trial RTI; respiratory tract infection; URTI; upper respiratory tract infection; RR: risk ratio.

**Table 2:** Probiotic clinical trials investigating respiratory infection etiology.

Study Type and Reference	Randomized Subjects	Probiotic Intervention	Analyzed Viruses	Study Outcomes: Probiotic vs. Placebo
Community				
R DB PC [81]	94 pre-term infants (2 days–2 months)	*L. rhamnosus* GG 1 × 10^9^ CFU/day (1–30day) and 2 × 10^9^ CFU/day for 31–60 days or galacto-oligosaccharide or placebo for 60 days	From nasal swab: − Human bocavirus − Rhinovirus/Enterovirus − RSV A and B − Adenovirus − Coronaviruses types 229E/NL63, OC43/HKU1 − Influenza A and B virus − Human metapneumovirus − PIV 1–3	Lower incidence of rhinovirus-induced RTI episodes (*p* = 0.04).
				Lower number of rhinovirus findings in acute RTI over 12 months (*p* = 0.015).
				No significant difference in the mean duration of symptoms in rhinovirus episodes, severity scores of clinical symptoms in rhinovirus episodes, rhinovirus RNA load during infections, duration of rhinovirus RNA shedding, duration or severity of rhinovirus.
R DB PC [84]	269 otitis-prone children (9 months–5.6 years)	*L. rhamnosus* GG, *L. rhamnosus* Lc705, *B. breve* 99 and *Propionibacterium jensenii* JS 8–9 × 10^9^ CFU/day of each strain, or placebo in a capsule for 6 months	From nasal swab: − Human bocavirus 1–4 − Rhinovirus/Enterovirus	Lower number of human bocavirus 1 positive sample during the study (6.4% vs. 19.0%, *p* = 0.039).
				No effect on rhinovirus/enterovirus occurrence.
R DB PC [83]	97 daycare children (2–6 years) visiting health care practitioner due to RTI	*L. rhamnosus* GG approximately 10^8^ CFU/day in milk for 28 weeks	From nasal swab: − Human bocavirus 1–4 − Rhinovirus/Enterovirus − RSV − Adenovirus − Influenza A virus − PIV 1–2	Children had less days with respiratory symptoms per month (6.5 vs. 7.2, *p* < 0.001).
				No effect on the occurrence of respiratory viruses during the study or respiratory symptoms associated with viral findings.
R DB PC [85]	192 military conscripts (18–30 years) visiting health care practitioner due to RTI	*L. rhamnosus* GG 5 × 10^9^ CFU/day + *B. lactis* BB-12 2 × 10^9^ CFU/day in a chewing tablet for either 3 or 6 months	From nasal swab: − Human bocavirus − Rhinovirus/Enterovirus − RSV A and B − Adenovirus − Coronaviruses types 229E/NL63, OC43/HKU1 − Influenza A and B virus − Human metapneumovirus − PIV 1-4	Overall no significant effect on the occurrence of common respiratory viruses. In a subgroup, there was lower occurrence of rhino/enteroviruses after 3 months (5 vs. 15, *p* < 0.01).
Open label, parallel group [82]	2926 schoolchildren (6–12 years)	*L. brevis* KB290 in a nutrient drink 6 × 10^9^ CFU/bottle 5 day/week for 8 weeks + no consumption for 8 weeks or vice versa (2 alternate study groups)	Physician diagnosed influenza virus infection	During influenza epidemic, less influenza infections in the group consuming probiotic drink compared with the group not consuming probiotic drink (15.7% vs. 23.9%, *p* < 0.001)
R DB PC [86]	209 nursing home residents aged ≥65 years	*L. rhamnosus* GG 2 × 10^10^ CFU/d in capsule or placebo for 6 months	From nasal swab: − Rhinovirus/Enterovirus − RSV − Influenza A and B virus − Human metapneumovirus − PIV 1–3	No statistically significant difference in laboratory confirmed viral respiratory infections.
Experimental Virus Challenge				
R DB PC [87,88]	59 healthy adults (mean 22–24 years)	*L. rhamnosus* GG 10^9^ CFU of live or heat-inactivated (by spray-drying) in 100 mL of fruit juice or control juice daily for 6 weeks.	From nasal lavage: − Rhinovirus A39	No significant effect on rhinovirus infection rate.
				No significant effect on the occurrence and severity of cold symptoms during rhinovirus infection.
				No significant effect on viral loads.
R DB PC [89]	115 healthy adults with confirmed experimental infection (mean 22–23 years)	*B. lactis* Bl-04 2 × 10^9^ CFU powder or placebo daily for 32 days	From nasal lavage: − Rhinovirus A39	Reduction in nasal rhinovirus titer and the proportion of subjects shedding virus in nasal secretions (76% vs. 91%, *p* = 0.04) during the infection.
				Significantly higher IL-8 levels in nasal lavage prior to infection (90 vs. 58 pg/mL, *p* = 0.04). Significantly reduced IL-8 response to rhinovirus infection in nasal lavage (*p* = 0.03).
				No significant effect on symptom severity/scores or infection rate.

Abbreviations: CFU: colony forming unit; PIV: parainfluenza virus; R DB PC: randomized double-blind placebo-controlled; RSV: respiratory syncytial virus; RTI: respiratory tract infection.
